# PReS-FINAL-2339: Blocking interferon alpha signaling can reduce neutrophil extracellular trap formation in juvenile onset systemic lupus erythematosus

**DOI:** 10.1186/1546-0096-11-S2-P329

**Published:** 2013-12-05

**Authors:** A Midgley, MW Beresford

**Affiliations:** 1 Department of Women's and Children's Health, University of Liverpool, Liverpool, UK

## Introduction

A novel antimicrobial mechanism of neutrophils involves the release of neutrophil extracellular traps (NETs) into the local environment to bind pathogens. NETs are composed of chromatin, and it has been proposed that a source of autoantigens in Systemic Lupus Erythematosus (SLE) could be increased NETs production. Interferon (IFN)-α is known to be a key player in the pathogenesis of SLE. In Juvenile-onset SLE (JSLE) patients with generally more severe disease, an IFN signature is almost invariably shown at early stages of disease, suggesting that activation of the type-I IFN pathway may be especially important in the induction of the disease process.

## Objectives

Observe and measure the induction of NETs in JSLE patients and controls following incubation with IFN alpha and serum ± pre incubation with an inhibitor to block IFN signalling.

## Methods

Neutrophils isolated from children with JSLE and controls were either left unstimulated, pre-incubated with IFN alpha for 30 mins or incubated for 2 hours with 10% JSLE or control serum, 300 nm PMA, and 100 ng IFN-alpha. Induction of NETs was visualised using confocal microscopy following staining for Neutropil Elastase, dsDNA and DAPI. Pre incubation with 10uM JAK inhibitor was used to inhibit IFN alpha signalling. The IFN alpha environment of the JSLE and control neutrophils prior to isolation was measured using pSTAT1 and western blotting. To quantify NETs formed cultured neutrophils were digested using micrococcal nuclease to dismantle the NET scaffold, double stranded DNA was quantified using the Quant-iT Picogreen assay. Extracellular DNA was measured using a spectrofluorometer.

## Results

Formation of NETs were observed following incubation with PMA, JSLE serum and IFN-alpha (confocal microscopy images). Unstimulated neutrophils and neutrophils incubated with control serum were not seen to form NETs. Greater NET formation was observed in JSLE neutrophils incubated with JSLE serum and IFN-alpha compared to control neutrophils. Increased pSTAT1 signalling was observed in JSLE (0.9 ± 0.05) compared to control neutrophils (0.42 ± 0.08, p < 0.05). Pre-incubation of control neutrophils with IFN alpha increased amount of NETs induced. NET formation following incubation with JSLE serum subsequently lead to the exposure of dsDNA (confocal microscopy images). NETs induced by JSLE serum and IFN alpha was reduced following pre incubation with a JAK inhibitor.

## Conclusion

This study observed the induction of NETs in JSLE and controls neutrophils and that control and to a greater extent JSLE neutrophils form NETs following incubation with JSLE serum and IFN-alpha. This led to exposure of dsDNA therefore providing further evidence that NET formation could be a potential source of autoantigen exposure in SLE. The results from our study suggest that the formation of NETs in JSLE may be exacerbated by the factors expressed in JSLE serum including IFN alpha. Blocking of IFN alpha signalling using a JAK inhibitor resulted in reduced NET formation as observed by confocal microscopy and quantification of DNA following digestion of NETs. The JAK inhibitor, Tofacitinib has recently been approved as a treatment for Rheumatoid Arthritis, this study may provide evidence that JAK inhibitors may be a useful therapeutic agent in patients with SLE.

## Disclosure of interest

None declared.

